# Frequencies of CD8 and DN MAIT Cells Among Children Diagnosed With Type 1 Diabetes Are Similar to Age-Matched Controls

**DOI:** 10.3389/fimmu.2021.604157

**Published:** 2021-02-23

**Authors:** Robert Z. Harms, Katie R. Ostlund, Monina Cabrera, Earline Edwards, Victoria B. Smith, Lynette M. Smith, Nora Sarvetnick

**Affiliations:** ^1^Department of Surgery-Transplant, University of Nebraska Medical Center, Omaha, NE, United States; ^2^Pediatric Endocrinology, University of Nebraska Center, Omaha, NE, United States; ^3^Children's Pediatric Endocrinology, Children's Hospital and Medical Center, Omaha, NE, United States; ^4^Office of the Vice Chancellor of Research, University of Nebraska Medical Center, Omaha, NE, United States; ^5^Department of Biostatistics, University of Nebraska Medical Center, Omaha, NE, United States; ^6^Mary and Dick Holland Regenerative Medicine Program, University of Nebraska Medical Center, Omaha, NE, United States

**Keywords:** mucosal associated invariant T cells, type 1 diabetes, autoantibodies, vitamin D, human cytomegalovirus, interleukin 18, interleukin 7

## Abstract

Mucosal-associated invariant T (MAIT) cells have been implicated in various forms of autoimmunity, including type 1 diabetes (T1D). Here, we tested the hypothesis that CD8 and double negative (DN) MAIT cell frequencies were altered among diagnosed T1D subjects compared to controls. To do this, we analyzed cryopreserved peripheral blood mononuclear cells (PBMCs) from age-matched T1D and control children using flow cytometry. We observed that CD8 and DN MAIT cell frequencies were similarly abundant between the two groups. We tested for associations between MAIT cell frequency and T1D-associated parameters, which could reveal a pathogenic role for MAIT cells in the absence of changes in frequency. We found no significant associations between CD8 and DN MAIT cell frequency and levels of islet cell autoantibodies (ICA), glutamate decarboxylase 65 (GAD65) autoantibodies, zinc transporter 8 (ZNT8) autoantibodies, and insulinoma antigen 2 (IA-2) autoantibodies. Furthermore, CD8 and DN MAIT cell frequencies were not significantly associated with time since diagnosis, c-peptide levels, HbA1c, and BMI. As we have examined this cohort for multiple soluble factors previously, we tested for associations between relevant factors and MAIT cell frequency. These could help to explain the broad range of MAIT frequencies we observed and/or indicate disease-associated processes. Although we found nothing disease-specific, we observed that levels of IL-7, IL-18, 25 (OH) vitamin D, and the ratio of vitamin D binding protein to 25 (OH) vitamin D were all associated with MAIT cell frequency. Finally, previous cytomegalovirus infection was associated with reduced CD8 and DN MAIT cells. From this evaluation, we found no connections between CD8 and DN MAIT cells and children with T1D. However, we did observe several intrinsic and extrinsic factors that could influence peripheral MAIT cell abundance among all children. These factors may be worth consideration in future experimental design.

## Introduction

In human populations across the globe, modern living conditions have been associated with increased incidence and prevalence of various forms of autoimmunity, including type 1 diabetes (T1D) (1, 2). Several causal factors are hypothesized to account for this association. Of these, intestinal dysbiosis is particularly provocative when considering the etiology of T1D. This etiological theory is based upon microbial population dynamics in the gut lumen. Pathogenic microbial imbalances are thought to lead to mucosal inflammation and enterocyte damage, and, ultimately, to increased exposure to microbial products and infectious agents. Such exposure has been postulated to promote the breakdown of immunological tolerance, as well as negatively impact beta cell health by creating an inflammatory environment in and around the pancreatic islets (3, 4). Evidence for this theory includes histological findings of increased inflammation in the intestinal mucosa, elevated circulating zonulin, as well as several reports of distinct bacterial floras associated with T1D (5–11).

The mucosal interface of the gastrointestinal system is a region of vigorous communication between microbial visitors and the host. There, an extensive array of immunological crosstalk occurs, serving to modulate the environment for the benefit of prospective denizens or the detriment of opportunistic invaders. Among the many contributors to this conversation are mucosal-associated invariant T (MAIT) cells. Capable of directly lysing bacterially-infected targets and producing the proinflammatory cytokines IFN-γ, TNFα, and IL-17, MAIT cells are uniquely situated within the T cell lineage (12–14). Their distinct functions are partially mediated through a semi-invariant T cell receptor, which limits their antigen responsiveness to MR1-expressing cells harboring microbially-derived vitamin B metabolites (15, 16). In addition to their established role in microbial immunity, MAIT cells have been implicated in the etiologies of multiple autoimmune diseases (17).

A critical challenge in the study of T1D is the relative inability to directly examine tissues of interest in the affected population, here being intestine and pancreas. From this impasse, immunological studies of human T1D are largely relegated to venous blood and circulating leukocytes. As the majority of immune cells migrate through the vascular system with some regularity, it's possible to isolate collections at any given time to generate a profile of immune cell maturity, activation, and responsiveness. Interpretation of the results obtained from these studies is often challenging, resulting from the plethora of variables that can influence immune cell subsets (18).

Recently, we completed an analysis of peripheral blood mononuclear cells examining T cell subsets for individuals at-risk for type 1 diabetes (19). Among several intriguing observations, we found a reduction in frequency of MAIT cells in first- and second-degree relatives of subjects diagnosed with T1D. These relatives had also tested positive for the presence of autoantibodies. Yet, upon stratifying these individuals into those who progressed to disease and those who did not, we found the MAIT cell decrease was limited to those who did not progress to disease. To complement and extend our TrialNet study, we performed a similar analysis on children diagnosed with T1D and healthy controls obtained locally. Our goal was to compare T cells pre-and post-disease, to get a clearer picture of any immunological processes that may be observable in either state. We hypothesized that MAIT cell frequencies would be changed in T1D, due to the effects of reported intestinal dysbiosis and a previous report demonstrating reduced MAIT cell frequencies in juvenile T1D subjects (20). Although our results did not support this hypothesis, we did make several observations that may help to explain the peripheral abundance of MAIT cells.

## Methods

### Patients

This cohort has been described previously (21) and consists of 27 control (no history or family history of autoimmunity, short-stature diagnosis) and 36 diagnosed juvenile T1D subjects. Subjects' ages range from 8 to 11 years old. Complete patient data are available in the aforementioned publication. Of note, we detected low “positive” levels of GAD65, ZNT8, or IA-2 autoantibodies in some control subjects using commercially-available solid phase assays. Conversely, no control subject was positive for ICA. These positive autoantibody measurements could represent random presence of autoantibodies in healthy subjects as others have reported (22–26), and, thus, a potential sign of risk for developing T1D. Importantly, these measurements were from research use only (RUO) assays, rather than the clinically-diagnostic assays that were used to diagnose the T1D subjects upon disease presentation. Furthermore, we were unable to pursue these subjects longitudinally to confirm these measurements as either fleeting or persistent. Thus, while we are reluctant to describe these subjects as “at-risk” of developing T1D, we consider the issue of enough interest to include correlations of these autoantibody levels with MAIT cell frequency below.

Subjects were enrolled from the Endocrine Clinic at Children's Hospital and Medical Center in Omaha. The study was approved by the University of Nebraska Medical Center Institutional Review Board (IRB# 107-09-EP) and performed in accordance with 1964 Declaration of Helsinki. Parental/guardian permission and child assent was obtained for all participants.

### Sample Processing

We acquired 20 mL of venous blood in K2 EDTA vacutainers (Becton, Dickinson, and Company). Following the isolation of plasma, the remaining whole blood was diluted in PBS, overlayed onto Ficoll-Paque Plus (GE Healthcare), and centrifuged. Peripheral blood mononuclear cells were isolated from the interface, then washed and centrifuged 3 times to reduce platelet abundance. Cell pellets were then resuspended in RPMI 1640 (HyClone™) supplemented with 10% v/v fetal bovine serum (HyClone™), 2 mM L-glutamine, 25 mM HEPES, 50 IU penicillin and 50 μg/mL streptomycin (Corning). We then slowly added an equal volume freezing media (fetal bovine serum + 20% v/v dimethysulfoxide (Fisher BioReagents™) with gentle mixing. Aliquots of PBMCs were then placed in Nalgene “Mr. Frosty” freezing containers frozen at −80°C overnight. The following morning, cell aliquots were transferred to a −150°C cryogenic chest freezer for long term storage.

### Flow Cytometry

Samples were thawed rapidly with swirling in a 37°C water bath. They were then washed 3 times with X-Vivo 15 (Lonza) supplemented with 2% v/v human AB serum (MP Biomedical). Viability and count was confirmed with a hemocytometer and trypan blue. PBMCs were plated into pre-warmed, equilibrated media and rested overnight at 37°C with 5% CO_2_. The next morning, PBMC were recovered and again counted and tested for viability as above. Cells were then distributed to 96-well plates for surface staining. Surface staining was conducted as described previously (19). The following antibodies and reagents were used: PE CCR7 (G043H7), PE-Cy7 CCR4 (L291H4), BV711 CD14 (M5E2), BV711 CD19 (HIB19), BV785 CD27 (O323), FITC CD57 (HCD57), PerCP-Cy5.5 CD45RA (HI100), BV605 Vα7.2 (3C10), AF700 CD45 (HI30), and Streptavidin-APC from BioLegend; BV421 CXCR5 (RF8B2), BUV737 CD3 (UCHT1), BUV395 CD4 (RPA-T4), BV510 CD8 (RPA-T8), PE-CF594 CD28 (CD28.2), BV650 CD161 (DX12) from BD Biosciences; Biotin CD127 (eBioRDR5) from eBioscience; LIVE/DEAD™ UV Blue from Molecular Probes. Following antibody labeling and fixation, samples were analyzed on a 5 laser LSR II (Becton, Dickinson and Company) within 24 h. Flow cytometry data was analyzed using FlowJo™ v 10.2 (Becton, Dickinson and Company).

### ELISA

The ELISA methodology, procedures, and outcomes were described fully previously (21). Briefly, Vitamin D BP (DVDBP0) and high sensitivity IL-7 (HS750) assays were acquired from R&D Systems; the 25 (OH) Vitamin D assay (ADI-900-215) was acquired from Enzo BioChem; IL-18 (BMS267) from ThermoFisher; GAD65 (GAD31-KO1), ICA (ICA31-KO1), ZNT8 (ZT831-KO1), and IA-2 (IA231-KO1) were acquired from Eagle Biosciences; CMV IgG (EG-101) acquired from IBL America; the c-peptide assay (80-CPTHU-E01.1) was acquired from Alpco. C-peptide values are from random, non-fasting measurements. Optical density (OD) measurements were taken using a BioTek H2 Hybrid plate reader and standard curves were generated with the associated Gen5 software v 3.05.11 (BioTek). Coefficient of infectivity (COI) for CMV is calculated according to manufacturer's recommendation and is the OD of the sample divided by the OD of the cut off control. Samples that possessed a COI that was greater than the COI + 10% were considered seropositive for CMV IgG.

### Statistics

We normalized values with natural log transformations and then tested for significant differences using the two-tailed Student's *t*-test for pairwise comparisons. Pearson's product moment correlation test on normalized values was used for correlations. Square root transformations were used for time since diagnosis correlations, as these values included true zeroes. Results were significant if *p* < 0.05. For analysis of how circadian rhythms may influence MAIT cell frequency, blood collection times were recorded using a 24 h clock, with a “military” -style notation. For example, 1:30 p.m. would be 1330. Univariate logistic regression and receiver operator curves (ROC) were used to examine the markers as predictors of T1D vs. control. The area under the ROC curves (AUC) along with a 95% confidence intervals were used to evaluate the predictive ability of the markers. Statistical tests were performed using SPSS v 25 (IBM), Prism v 6.03 (GraphPad Software), and SAS v 9.4 (SAS Institute Inc., Cary, NC). Figures were constructed using Prism and R (27).

## Results

### Frequencies of CD8 and DN MAIT Cells Are Similar Among Controls and T1D Subjects

**Figure 1:**
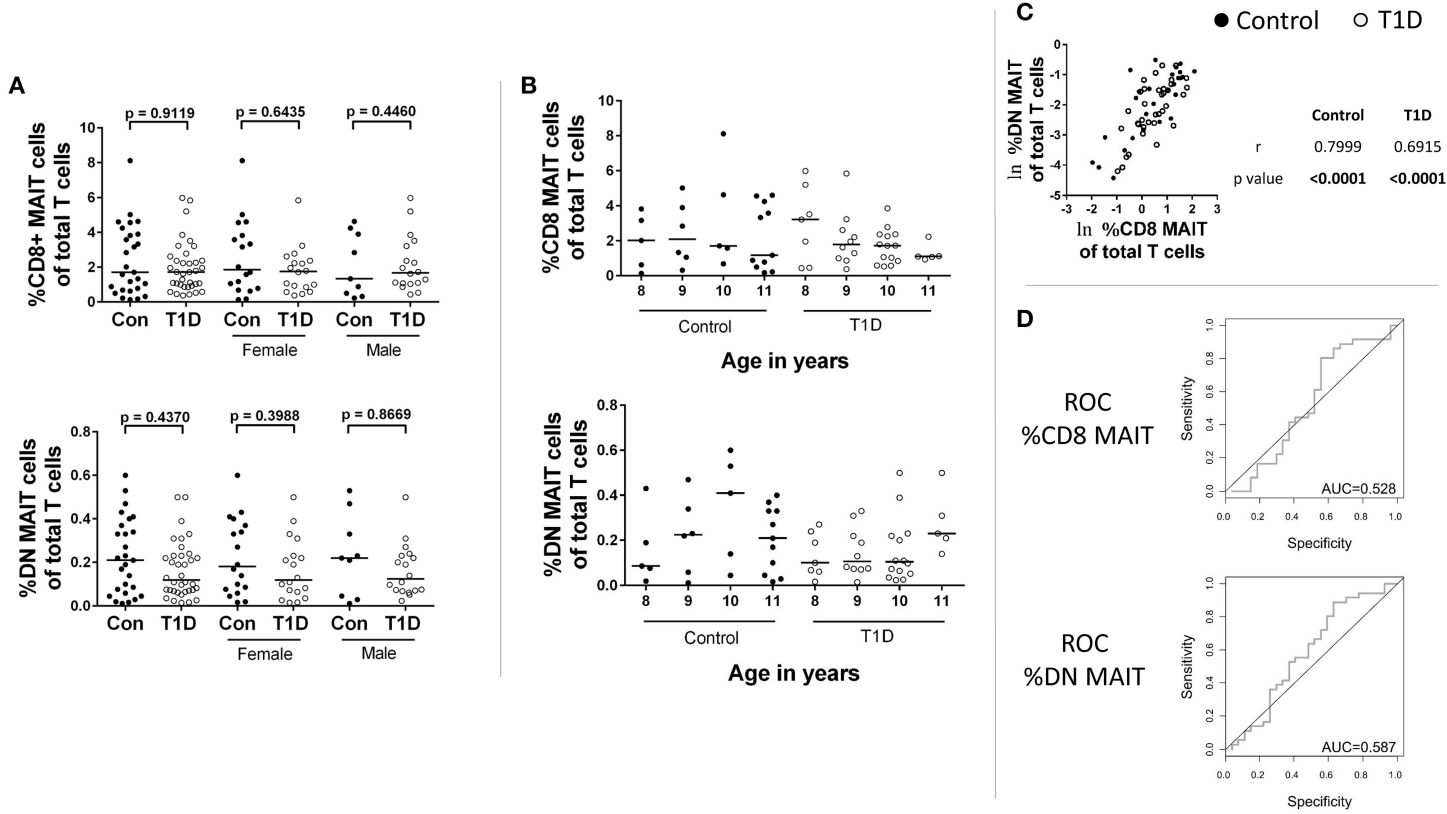
CD8 and DN MAIT cell frequencies among control and T1D subjects. **(A)** Frequency of CD8 MAIT cells and DN MAIT cells of total T cells among control and T1D subjects, as well as stratified by sex. **(B)** Subjects were divided according to their age in years to allow for visual examination of differences. **(C)** Correlation between CD8 MAIT cell frequency and DN MAIT cell frequency among T1D and control subjects. **(D)** Receiver operating characteristic (ROC) curves for CD8 and DN MAIT cell frequencies as predictors of T1D compared to control. For graphs in **(A,B)**, bars are median values. For **(C)**, ln denotes that values were transformed using natural logarithm. AUC, area under the curve.

The phenotypic identification of MAIT cells in peripheral blood and their stratification into CD8 and double-negative (DN) subsets was identical to what we reported previously (19). A representative gating strategy and data depiction is presented in Supplementary Figure 1. When comparing juvenile T1D subjects with controls, we observed no significant changes in frequency of either CD8 or DN MAIT cells (Figure 1A and Table 1). Furthermore, no differences were observed upon stratifying the subjects by sex (Figure 1A). Notably, we found a fairly broad range of values for MAIT cell frequencies among both T1D and control subjects (Table 1). Among controls, frequencies of CD8 MAIT cells ranged from 0.14 to 8.12% (58x) and DN MAIT cells ranged from 0.012 to 0.6% (50x). For T1D subjects, frequencies of CD8 MAIT cells ranged from 0.38 to 5.99% (15.8x) and DN MAIT cells range from 0.015 to 0.5% (33.3x). Although our cohort was tightly age-matched, stratifying subjects by age revealed these wide ranges were not overly influenced by age (Figure 1B). Rather, a wide range of values could be observed at almost any given year of age.

**Table 1:** Descriptive statistics of CD8 and DN MAIT cell frequency among total T cells.

	**%CD8 MAIT of total T cells**		**%DN MAIT of total T cells**	
	**Control**	**T1D**	**Control**	**T1D**
Mean	2.41	2.00	0.227	0.166
StdDev	2.00	1.46	0.174	0.129
Min	0.14	0.38	0.012	0.015
Max	8.12	5.99	0.600	0.500
Lower 95% CI	1.62	1.50	0.158	0.122
Upper 95% CI	3.21	2.49	0.296	0.209
Median	1.71	1.73	0.210	0.120

As we take a rather stringent view of MAIT cell phenotype (CD3+,Vα7.2+, CD45RA^low^, CD161^high^, CCR7^low^, CD127^high^, CD28^high^), we also examined less conservatively defined subsets for reference (see gating in Supplementary Figure 1). Similar to what we had already observed, results for CD3+, Vα7.2+, CD161^high^, CD45RA^low^ “total MAIT cells” and total Vα7.2+ T cells both failed to reveal significant differences in frequency between the two groups (Supplementary Figure 2).

The relationship between the CD8 and DN MAIT cell subsets is not completely clear. We were curious if relative frequency for the two subsets was similar per individual and if discrepancies associated with disease could be observed. Correlation results revealed the relative frequency of both subsets was significantly associated for both T1D and control subjects (Figure 1C).

Next, we asked whether CD8 MAIT cell and DN MAIT cell frequencies have any predictive value in assigning a type 1 diabetes diagnosis. We found that neither subset can be used to specifically and sensitively define T1D subjects in relation to control, thus appearing to have no predictive value (Figure 1D).

From these analyses, we found no evidence that MAIT cell frequencies are altered among juvenile T1D subjects, and they cannot be used to sensitively and specifically classify disease. In addition, wide-ranging frequencies are common in both control and T1D subjects even among age-restricted subjects.

### Frequencies of CD8 and DN MAIT Cells Are Not Associated With T1D-Related Parameters

**Figure 2:**
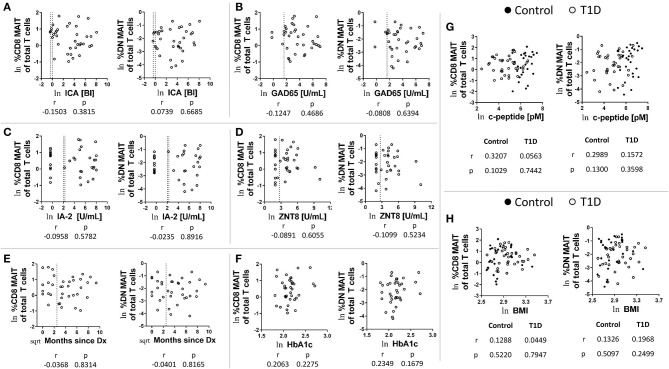
Correlations between CD8 and DN MAIT cell frequencies and T1D-associated parameters. Correlation between CD8 and DN MAIT cell frequency and **(A)** islet cell antibody (ICA) levels, **(B)** glutamic acid decarboxylase 65kD autoantibody (GAD65) levels, **(C)** insulinoma-associated antigen 2 (IA-2) autoantibody levels, and **(D)** zinc transporter 8 (ZNT8) autoantibody levels, **(E)** time since diagnosis, and **(F)** HbA1camong T1D subjects. Correlation between CD8 and DN MAIT cell frequency and **(G)** c-peptide level and **(H)** body mass index (BMI) among control and T1D subjects. For **(A–H)**, ln denotes that values were transformed using natural logarithm. For **(E)**, sqrt denotes values were transformed using square root transformation. For **(A–D)**, dotted lines represents manufacturer -defined cutoff values for designating positive or negative status for the given autoantibody. For **(E)**, dotted line represents 1 year from diagnosis. BI, binding index; U/mL, units per mL.

MAIT cells have been hypothesized to be capable of beta cell damage (20). Therefore, we investigated relationships among MAIT cell frequencies and several T1D-associated parameters. We found no significant correlations among MAIT cell frequencies and levels of autoantibodies for ICA, GAD65, IA-2, and ZNT8 (T1D subjects: Figures 2A–D; control subjects: Supplementary Figures 3A–D). Additionally, we found that MAIT cell frequencies were not correlated with time since diagnosis (Figure 2E), nor with HbA1c levels (Figure 2F). Although significantly reduced in T1D, c-peptide is detectable and is a direct link to beta cell function. We found no significant correlation among c-peptide levels and frequency of MAIT cells among T1D subjects nor among controls (Figure 2G). Lastly, we observed no significant relationship between BMI and MAIT cell frequency (Figure 2H), a relationship that has been previously reported (28). Similarly, we observed no significant correlation for BMI percentile (Supplementary Figure 3E). Combined, we found no connection between MAIT cell frequency and islet autoimmunity or T1D-associated metabolic variables.

### MAIT Cell Frequencies Are Associated With Levels of Circulating Vitamin D, IL-7, and IL-18, as Well as Previous CMV Exposure

**Figure 3:**
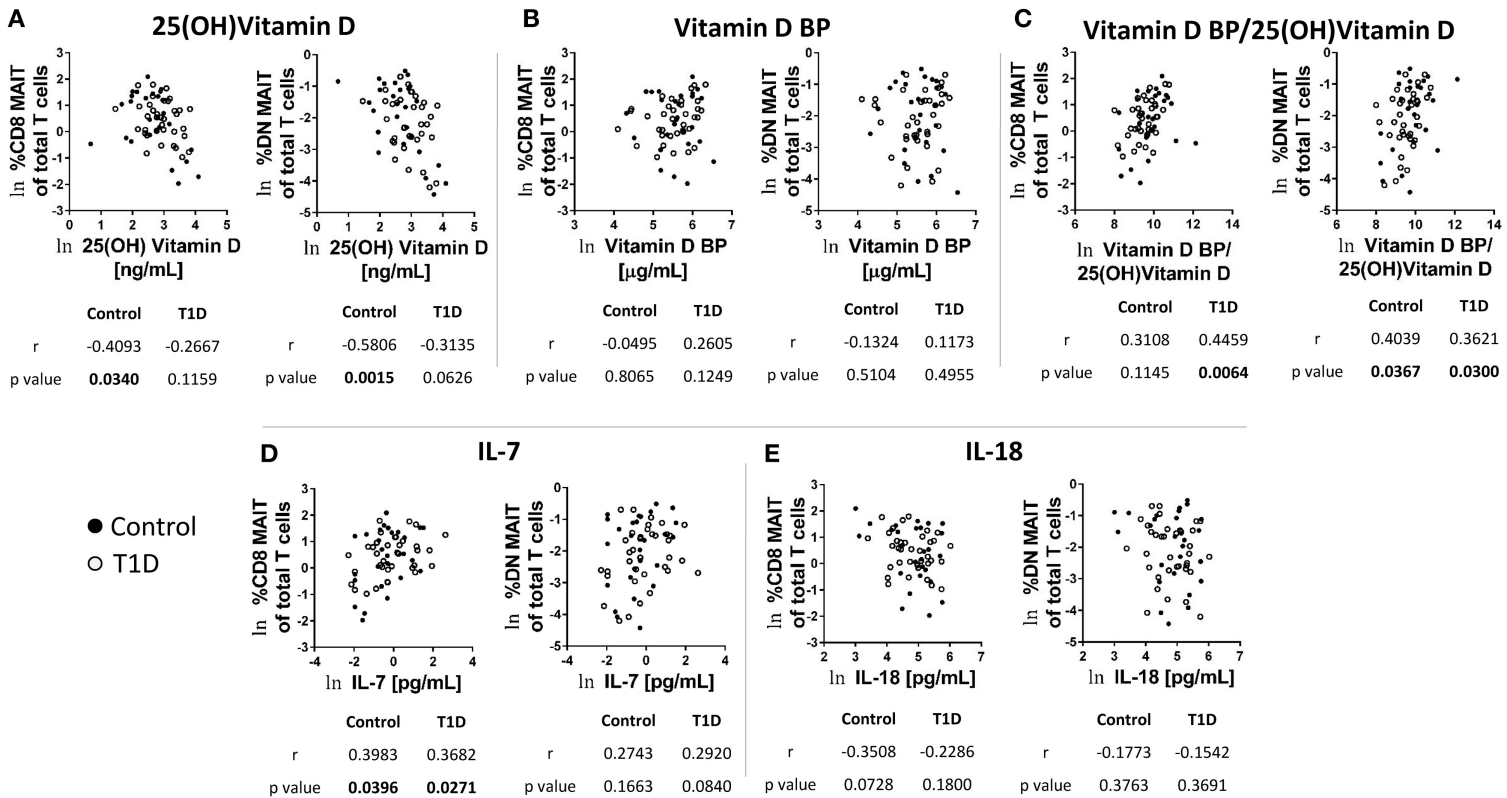
Correlations between CD8 and DN MAIT cell frequencies and select soluble factors in plasma. **(A)** Correlation between CD8 and DN MAIT cells frequency and 25 (OH) vitamin D **(B)** Correlation between CD8 and DN MAIT cells frequency and vitamin D binding protein (BP). **(C)** Correlation between CD8 and DN MAIT cells frequency and ratio of vitamin D BP to 25 (OH) vitamin D. **(D)** Correlation between CD8 and DN MAIT cells frequency and IL-7. **(E)** Correlation between CD8 and DN MAIT cells frequency and IL-18. For **(A–E)**, ln denotes that values were transformed using natural logarithm. Since these relationships **(A–E)** were more-or-less directionally similar, we also examined them by pooling the control and T1D subjects. These results can be viewed in Supplementary Figure 4.

As mentioned above, the frequency and abundance of circulating leukocytes is often difficult to interpret due to multiple variables contributing to frequency at a given time point (18). We have previously analyzed the plasma from these subjects for a wide range of soluble factors (21). Of these, a handful were pertinent to MAIT cell biology. These were vitamin D and its carrier, vitamin D binding protein, as well as the cytokines IL-7 and IL-18. We found that 25 (OH) vitamin D levels negatively correlated with both CD8 and DN MAIT cell frequencies (Figure 3A). These correlations were significant among controls, while appeared as trends among T1D subjects. As these results were directionally similar, we pooled all subjects together and observed significant negative correlations between 25 (OH) vitamin D and DN and CD8 MAIT cells (Supplementary Figure 4A). Thus, high levels of vitamin D are associated with lower levels of circulating MAIT cells. Vitamin D BP functions as a carrier molecule for Vitamin D. However, we observed no significant relationships among vitamin D BP and MAIT cells frequencies (Figure 3B). Lastly, we compared the ratio of vitamin D BP to vitamin D with MAIT cell frequencies. Such a ratio would query whether vitamin D bioavailability associates with MAIT cell frequency. Here, we observed significant positive correlations for CD8 MAIT among T1D subjects with a positive trend in among controls (Figure 3C). The ratio was similarly associated with DN MAIT cells among both controls and T1D subjects (Figure 3C). Again, we pooled both controls and T1D subjects and tested the relationship between the ratio and MAIT cell frequencies. We observed positive significant correlations (Supplementary Figure 4B). In total, these data suggest that when vitamin D is of limited bioavailability, MAIT cells frequencies are higher.

IL-7 is a cytokine involved in T cell homeostasis which also can directly influence MAIT cell function. In comparing IL-7 levels with MAIT cell frequencies, we observed significant positive correlations among CD8 MAIT cells for both T1D subjects and controls (Figure 3D). Similar trends were apparent for DN MAIT cells, but these did not reach significance (Figure 3D). Correlations among total pooled subjects revealed significant relationships for CD8 and DN MAIT cells (Supplementary Figure 4C). However, the strength of the association was stronger for CD8 MAIT cells. These data demonstrate that higher levels of circulating IL-7 are associated with greater MAIT cell frequencies.

The cytokine IL-18 promotes MAIT cell activation, and its abundance in circulation has been previously shown to be negatively associated with MAIT cell frequency among MS subjects (29). We tested correlations among MAIT cell frequency and IL-18 levels and observed negative trends, yet these did not reach significance (Figure 3E). We then tested the pooled subjects. We observed a significant negative correlation among IL-18 levels and CD8 MAIT cell frequency (Supplementary Figure 4D). Alternatively, the negative association among DN MAIT cells was not significant (Supplementary Figure 4D).

**Figure 4:**
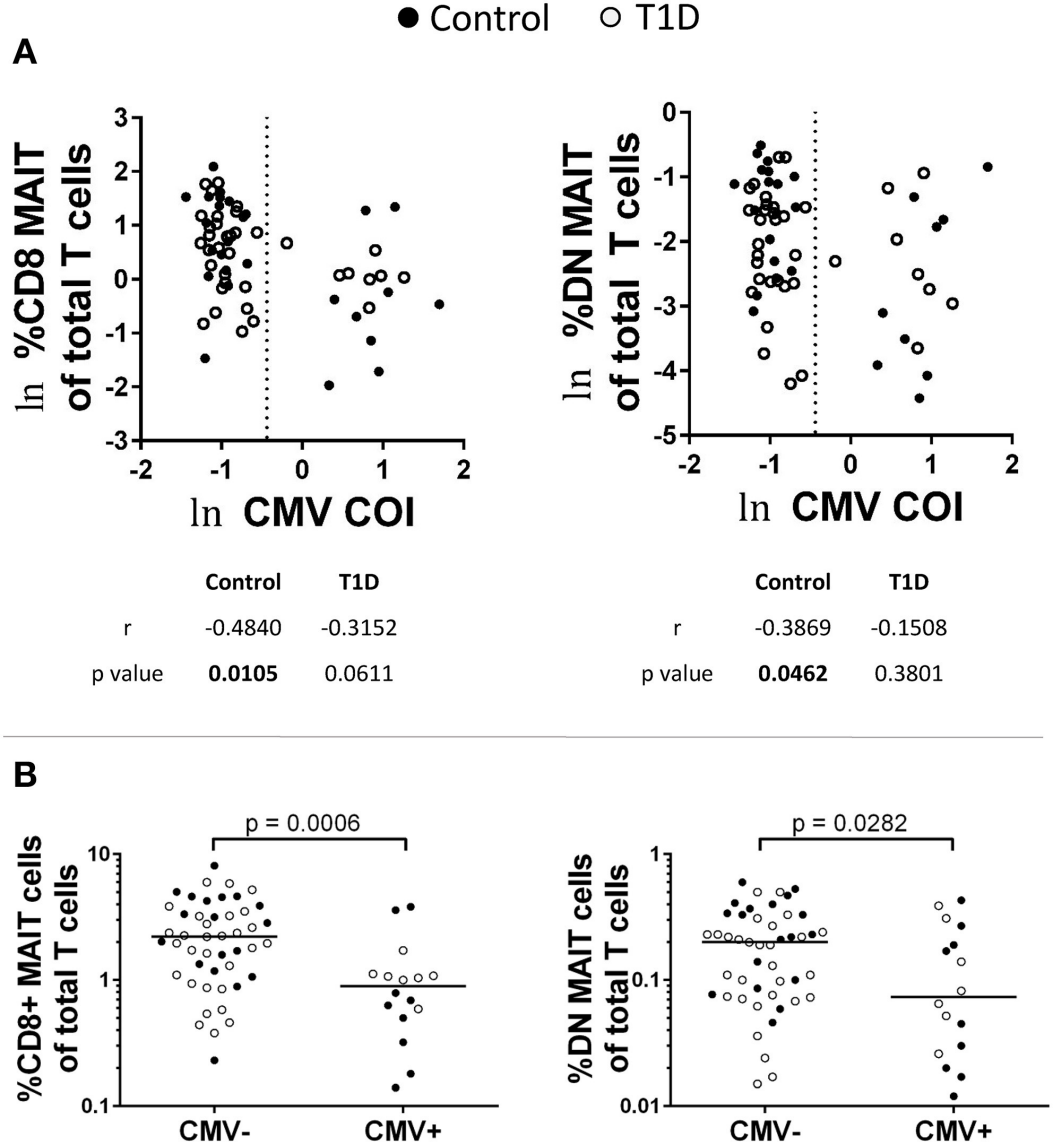
Influence of previous cytomegalovirus (CMV) infection on CD8 and DN MAIT cell frequencies. **(A)** Correlations between CD8 and DN MAIT cell frequencies and CMV cut off index (COI). **(B)** Frequencies of CD8 and DN MAIT cells among pooled T1D and control subjects that were stratified according to CMV seropositivity. For **(A)**, ln denotes that values were transformed using natural logarithm and dotted line represents cutoff point for determining CMV seropositivity.

Viral infection impacts MAIT cell abundance (30–35). Evidence suggests that this is due to indirect activation via cytokine signaling. To test whether or not MAIT cell frequencies were impacted by previous virus exposure, we tested for correlation with IgG levels for adenovirus, parainfluenza 1/2/3, Coxsackievirus, Epstein-Barr virus viral capsid antigen (EBV VCA), cytomegalovirus (CMV), and herpes simplex virus 1 (HSV1) (Figure 4A and data not shown). Of these, we observed significant negative correlation among MAIT cell frequency and CMV IgG abundance among controls, while T1D subjects approached significance for CD8 MAIT cells, yet were relatively weakly correlated for DN MAIT cells (Figure 4A). We then pooled and stratified our subjects into CMV+ and CMV– and compared MAIT cell frequencies. Among CMV+ subjects, CD8 and DN MAIT cells frequencies were lower than among CMV– subjects (Figure 4B). We observed no significant difference in frequency when stratified by disease into T1D or control (data not shown). From this, it appears that previous CMV infection may be associated with lower levels of MAIT cells.

Following these comparisons, we were curious if IL-18, IL-7, CMV, and vitamin D were associated with one another. Such associations could generate misleading results, for example, if all CMV-positive subjects possessed high amounts of vitamin D or highly reduced levels of IL-7. We compared each of these factors using correlative tests (Supplementary Figure 5). No association was significant, but the positive association between IL-7 and IL-18 levels approached significance (*p* = 0.0853). As we found that these two cytokines have opposite associations with MAIT cell frequencies, there may be a type of counterbalancing effect induced by the relative levels of the two factors.

Finally, circadian rhythms can influence lymphocyte abundance (18, 36). To our knowledge, there have been no reports on how time of blood collection is associated with MAIT cell frequency. Therefore, we examined CD8 and DN MAIT frequency in relation to time of blood draw. We observed no significant correlation among CD8 MAIT cells when comparing control or T1D groups individually, or when combined (Supplementary Figure 6A). Similarly, DN MAIT cells were not significantly correlated with time of blood draw among the control group or when combined (Supplementary Figure 6B). Interestingly, DN MAIT cell frequency in relation to time of blood draw approached significance among the T1D group (*r* = 0.2900 and *p* = 0.08; Supplementary Figure 6B). Although this may suggest a circadian relationship in DN MAIT cell frequency only among T1D subjects, such an unusual observation needs to be confirmed using a much larger cohort of T1D subjects and spanning multiple time points throughout the 24 h cycle. Indeed, our analysis was largely restricted to blood draws taken from 09:00 to 17:00. Only 1 subject was collected outside of this range, at time 07:55.

## Discussion

The relationship of T1D with intestinal health has a long history, due to pancreas adjacency/connectivity, and independent reports of mucosal damage associated with the disease. Being clearly-defined proinflammatory, microbial responders, MAIT cells could be relevant to T1D pathogenesis. A handful of investigations have sought to test this hypothesis by querying MAIT cell frequency, both before and following diagnosis (19, 20, 37, 38). The results of these independent observations are not entirely in agreement at either stage.

Among the investigations of juveniles following diagnosis, we previously observed a similar frequency and absolute number of CD8 MAIT-like cells among healthy control and T1D subjects (37). Importantly, frequency and absolute number were similar among T1D subjects classified as new-onset ( ≤ 1 year from dx) and long-standing (>1 year from dx). Following this, a reduction in MAIT cell frequency among T1D subjects ≤ 10 days from diagnosis was reported (20). It was also reported that these reductions persisted in a subset of these subjects for 1 year following diagnosis (ibid). In contrast, similar frequencies of MAIT cells were observed among healthy controls and T1D subjects <1 week following diagnosis (38). Likewise, here we found no change in CD8 or DN MAIT cell frequency among T1D subjects compared to healthy controls. These T1D subjects ranged in time from 0 to 88 months following diagnosis, but, unlike previous studies, were more age-restricted to avoid the known age-related bias associated with MAIT cell frequencies (37, 39, 40). With this experimental design, we observed no significant correlation between MAIT cell frequency and time since diagnosis, nor an indication that frequencies were reduced ≤ 1 year from diagnosis. Additionally, we observed no association between MAIT cell frequencies and autoantibody levels, c-peptide levels, HbA1c, and BMI. These results are in line with and complimentary to recent histological analyses of human pancreata which demonstrated no MAIT cell involvement in recently diagnosed T1D subjects (41). In total, from the known available literature, there is scant evidence that MAIT cells play a specific role in human T1D pathogenesis. Furthermore, there has been no confirmation that reduced MAIT cell abundance is a character state of T1D at any stage following diagnosis.

In the course of this investigation, we did make a handful of observations of utility in understanding MAIT cell biology. These could aid experimental design in future work. First, MAIT cell frequencies are widely variable even among age-restricted juveniles. While the totality of factors governing MAIT cell frequency is unknown, some have been explored here. There is likely also a genetic component similar to what is observed in murine biology (42). Second, CD8 and DN MAIT cell frequencies are tightly linked, suggesting that highly similar developmental and homeostatic forces dictate abundance of these two lineages. Recent comparative analysis of CD8 and DN MAIT cells has shown that the DN subset is somewhat less cytotoxic, less clonally diverse, and more prone to apoptosis than the CD8 subset (43, 44). Interestingly, our data indicates that the DN MAIT cell subset may be less responsive to cytokines than the CD8 subset. One explanation for this could be a reported reduced IL-18R expression by the DN MAIT cell subset (44).

From our analysis, we found that both intrinsic and extrinsic factors can influence MAIT cell abundance. Among the extrinsic factors, we found that subjects with the lowest levels of 25 (OH) vitamin D had the highest frequency of circulating MAIT cells. Complementary to this observation was that higher ratios of vitamin D BP to 25 (OH) vitamin D were associated with higher MAIT cell frequencies. Combined, these results suggest that the bioavailability of vitamin D influences MAIT cell abundance. Similarly, a recent report on vitamin D treatment among cystic fibrosis subjects found a tendency for MAIT cell reduction following a regimen of vitamin D supplementation (45). These observations indicate that sunlight exposure and dietary choice could directly impact MAIT cell frequency.

The mechanism whereby vitamin D influences MAIT cell frequency remains to be investigated. For direct effects, we were unable to find literature describing protein evidence of vitamin D receptor (VDR) expression by MAIT cells. However, a recent analysis reported a ~7 fold increase in VDR transcripts among stimulated MAIT cells compared to unstimulated (46). Assuming VDR is present in MAIT cells, higher vitamin D could limit their proliferation as has been shown for other T cells (47, 48). Indirectly, vitamin D can reduce antigen presentation and costimulation capabilities by human dendritic cells, monocytes, and macrophage (49, 50). The role of vitamin D in MR1 expression among APCs and epithelial cells is unknown and is worthy area of focused effort.

Intrinsically, both IL-7 and IL-18 have the ability to exert direct effects on MAIT cells as they express IL-7R and IL-18Rα. Several *in vitro* studies have shown that IL-7 contributes to MAIT cell effector function (51–53). On the other hand, IL-7 is well known to enhance proliferation and survival of human T cells (54) and a modest IL-7 proliferative potential has been shown among CD161^++^, IL-18Rα^+^, CD45RA^−^, CD27^+^ CD8 T cells (55). Furthermore, administration of rhIL-7 promotes CD8 T cell expansion (56, 57). Thus, it's likely that MAIT cell proliferation and abundance would be positively influenced in a high IL-7 environment. Similar to our findings, HIV+ subjects showed a positive relationship between IL-7 levels and MAIT cell frequency in the periphery (52). Thus, we confirm and extend this observation by noting the association among T1D subjects and healthy children.

Unlike IL-7, IL-18 levels were negatively associated with MAIT cell frequency. However, the associations were relatively weak, although somewhat stronger among CD8 MAIT cells than DN MAIT cells. Previous examinations of MS subjects and controls revealed a negative association between IL-18 levels and MAIT cell frequency among MS subjects, yet no such relationship among controls (29). In addition, multiple studies have demonstrated a role for IL-18 in driving MAIT cell activation (32, 58–60). Our inability to observe drastic effects associated with circulating IL-18 levels could have several explanations. One, there could be a scarcity of co-stimulating cytokines like IL-12p70 or IL-15 in the periphery. Alternatively, T1D subjects have been found to carry a SNP associated with reduced IL-18Rβ expression (61). Such reduced expression may limit IL-18 responses among MAIT cells. Lastly, circulating IL-18 is associated with the IL-18BP, whose levels are present in substantial excess of IL-18 in the plasma. This soluble inhibitor of IL-18 serves to lessen the effects of IL-18 (62). As our cohorts possessed relatively equivalent proportions of IL-18BP to IL-18 (21), similar, negligible effects from the cytokine could be expected between our two groups. However, the examination of IL-18 and MAIT cell frequency in scenarios when IL-18BP is diminished may lead to different results.

Due to our previous analysis of select viral immunoglobulins in these subjects (21), we were able to look for relationships between MAIT cell frequency and previous viral exposure. We observed that MAIT cells are less abundant in CMV+ subjects, irrespective of disease status. Other viruses examined, including two other herpesviruses, showed no such relationship. While these results are intriguing, we are approaching them with caution for two reasons. First, the CMV+ sample population consists of 16 children, which may be an improperly sized sample set for drawing extensive conclusions. Indeed, from the results reported here, the frequency range of MAIT cells can vary tremendously even among age-restricted children. Sampling bias in this scenario is certainly a possibility. Second, we examined CMV correlations with MAIT cell frequency previously and observed no relationship (19). Importantly, that analysis included subjects who spanned over 18 years of age, so such relationships may have been obscured by the change in MAIT frequency with age (37, 39, 40). To clarify this issue, additional, larger studies designed to investigate the question of human MAIT cell frequency in relation to chronic and latent infections are in order.

In summary, our examination of MAIT cell frequency in juvenile T1D subjects and controls failed to detect differences between the two groups. Furthermore, we found no significant associations among MAIT cell frequency and several disease-associated parameters. As it stands, we have found little evidence supporting a role for MAIT cells in human T1D. It should be noted, however, that our analysis did not include an examination of activation markers nor of soluble mediators. It could be that MAIT cells in T1D show signs of greater/lesser activation, or exhibit reduced/increased cytokine production. Also, our T1D subjects possessed a higher BMI than our control subjects. This may have confounded the results. Additionally, there is heterogeneity in age of onset in T1D (63). It could be that MAIT cells play a greater role in subjects who acquire the disease at earlier or later ages than those examined here. These and other questions, such as longitudinal dynamics of frequency and absolute number pre- and post-disease, remain critical.

Finally, our analysis revealed that circulating factors and previous CMV infection may influence MAIT cell frequency. In addition, wide-ranging frequencies among donors were commonly observed. In total, these factors, and several others known or awaiting discovery, may conceal or “create” differences between groups which could lead to erroneous interpretations. Future analyses of human MAIT cells should proceed with caution.

## Data Availability Statement

The original contributions presented in the study are included in the article/Supplementary Material, further inquiries can be directed to the corresponding author/s.

## Ethics Statement

The studies involving human participants were reviewed and approved by University of Nebraska Medical Center Institutional Review Board. Written informed consent to participate in this study was provided by the participants' legal guardian/next of kin.

## Author Contributions

RH and KO performed the experiments and analyzed data. RH processed samples. KO obtained consent and sample acquisition. MC and EE patient selection and sample acquisition. VS data acquisition. RH and LS statistical analysis. RH and NS experimental design. All authors contributed to the article and approved the submitted version.

## Conflict of Interest

The authors declare that the research was conducted in the absence of any commercial or financial relationships that could be construed as a potential conflict of interest.
