# Supplementary figures and images for: Frequencies of CD8 and DN MAIT Cells Among Children Diagnosed With Type 1 Diabetes Are Similar to Age-Matched Controls
Source: Front Immunol. 2021 Feb 23;12:604157. doi: 10.3389/fimmu.2021.604157 (PMC7940386; doi:10.3389/fimmu.2021.604157)

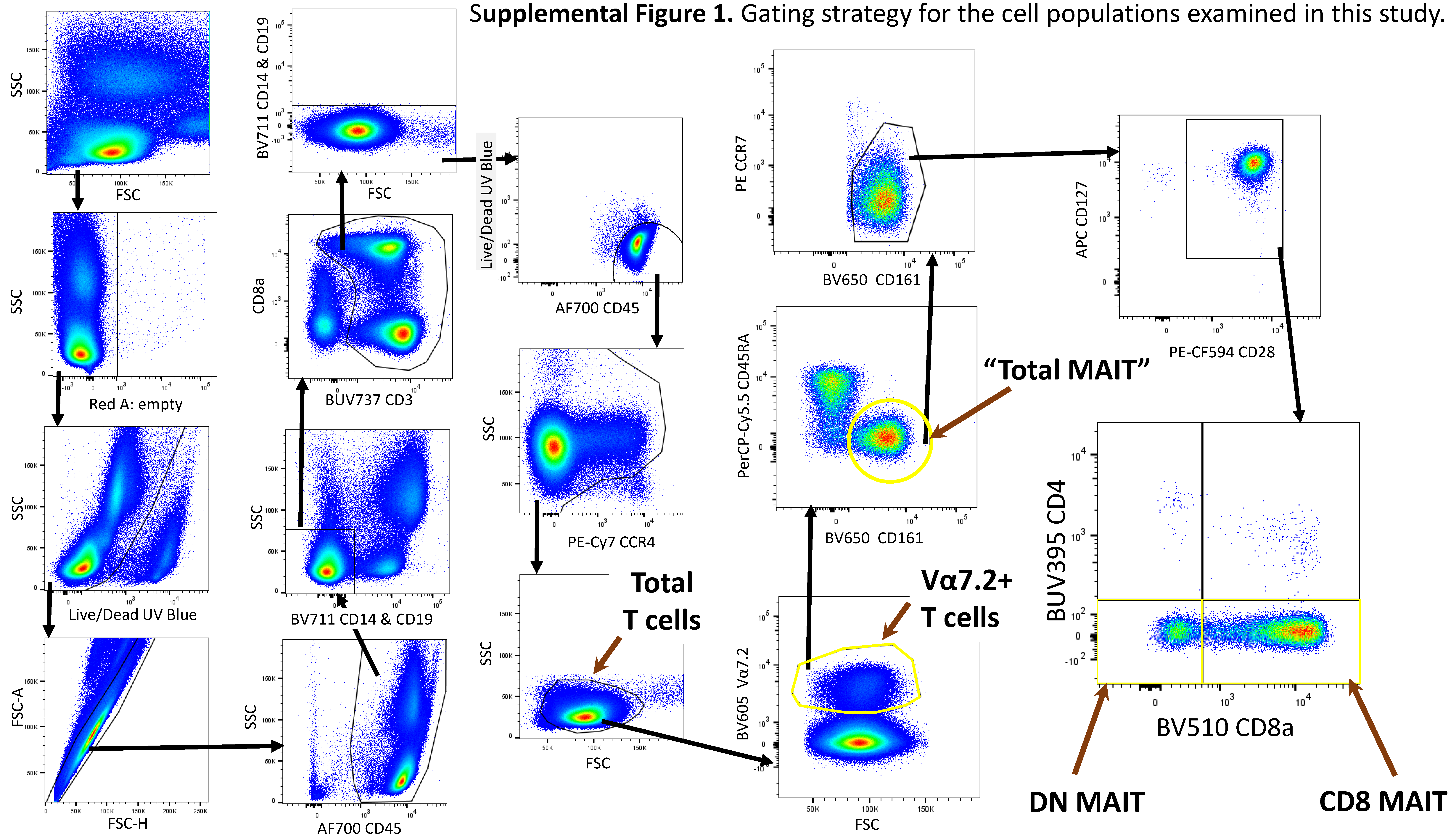

Supplement: Supplementary file 1 [file Image_1.TIF]

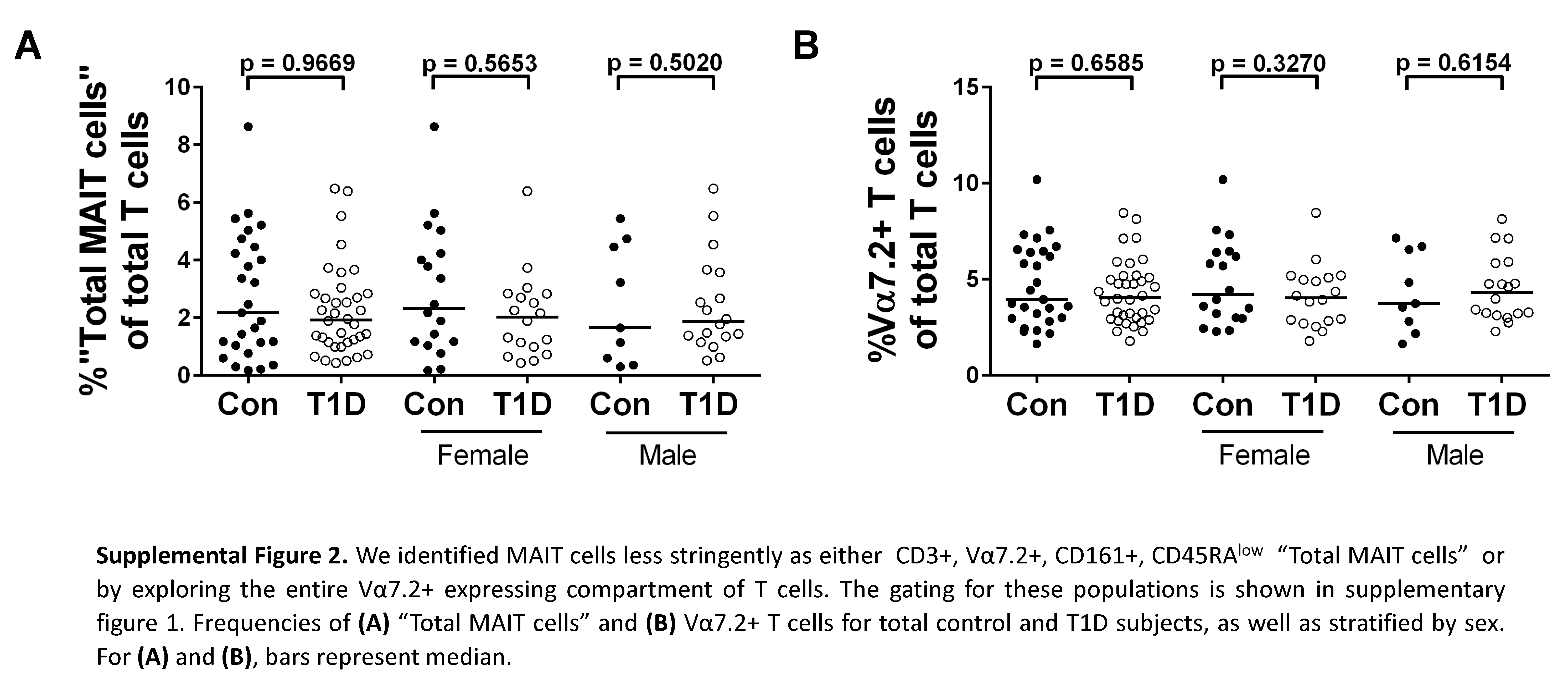

Supplement: Supplementary file 2 [file Image_2.tif]

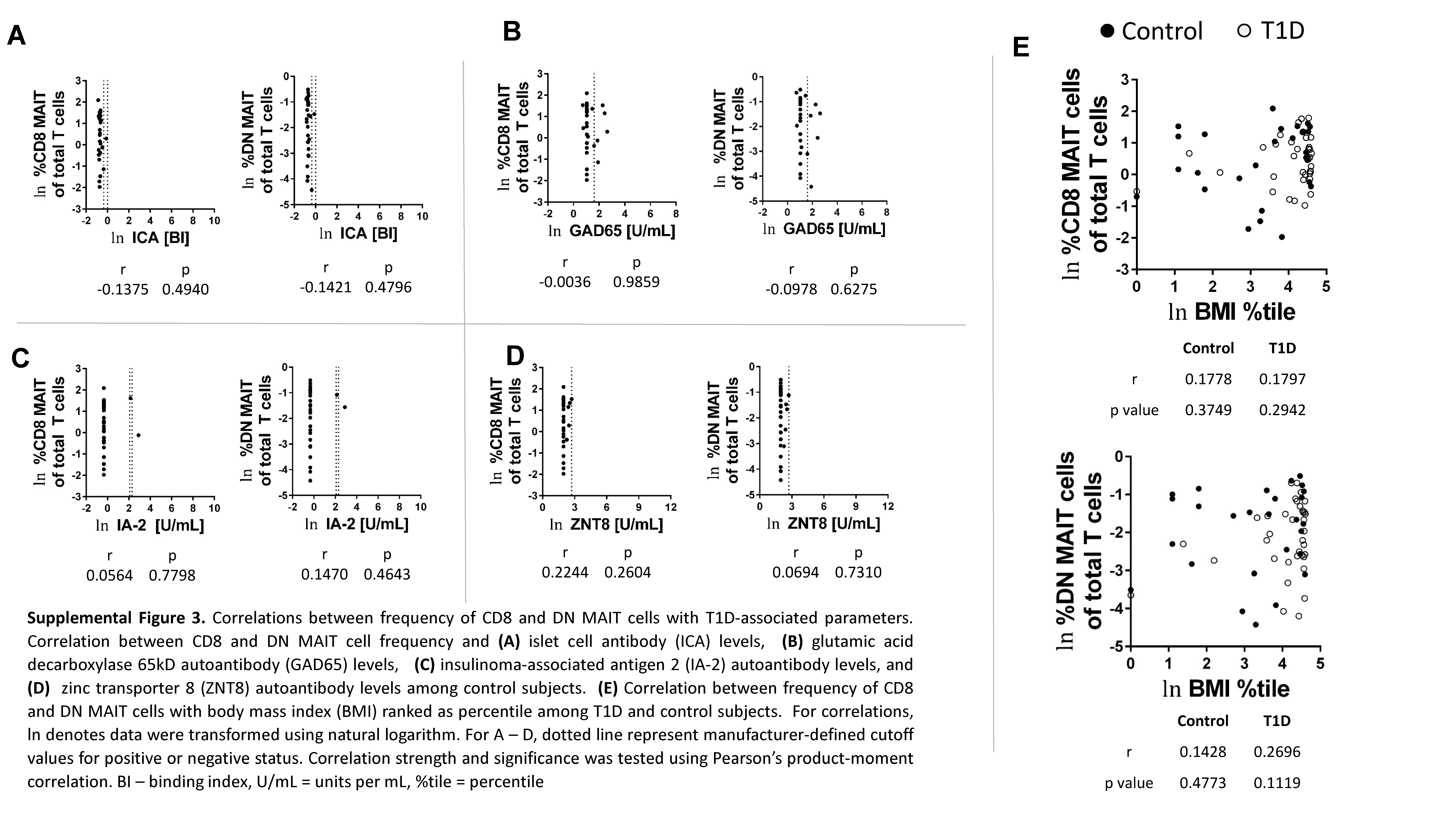

Supplement: Supplementary file 3 [file Image_3.TIF]

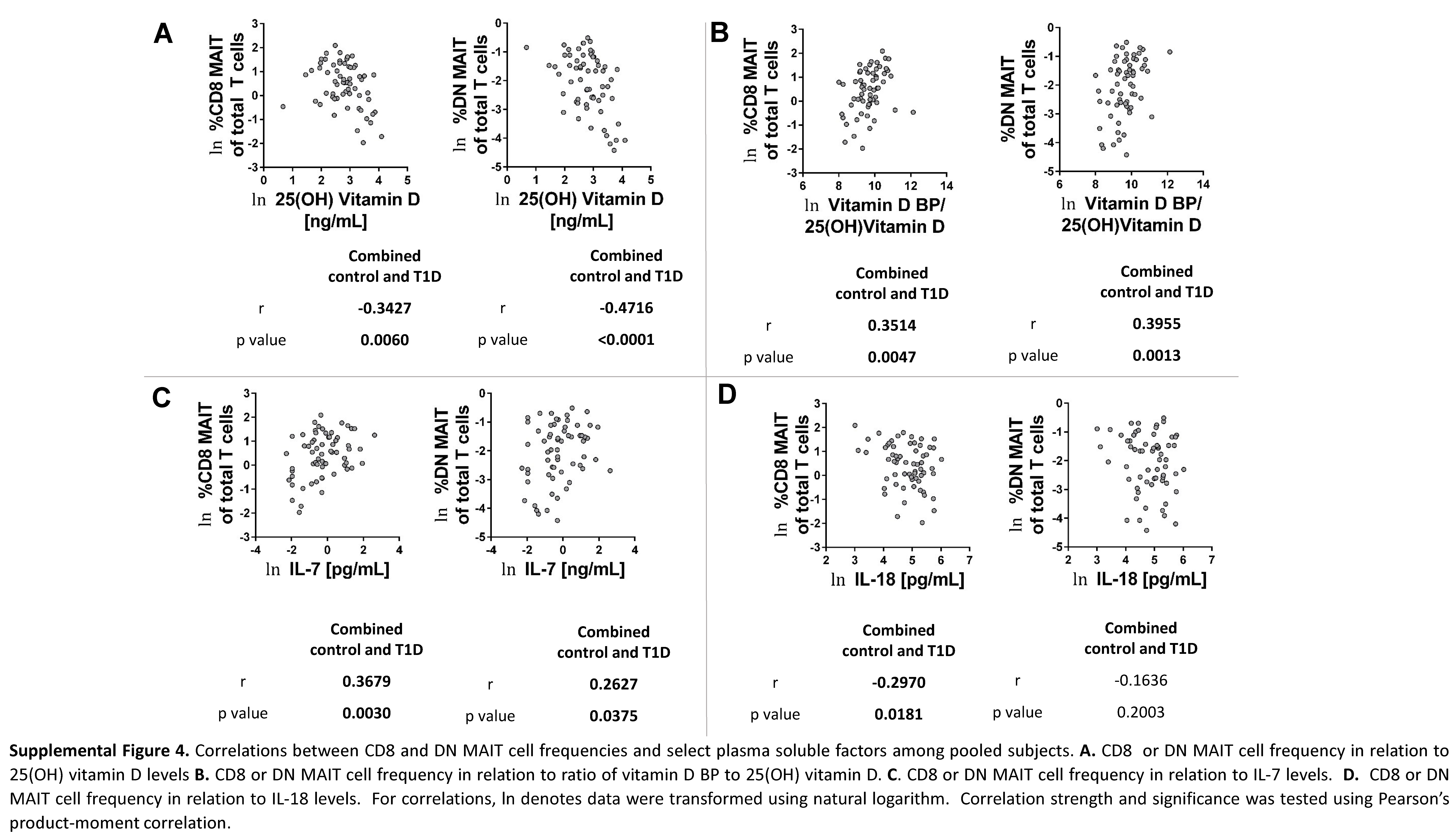

Supplement: Supplementary file 4 [file Image_4.TIF]

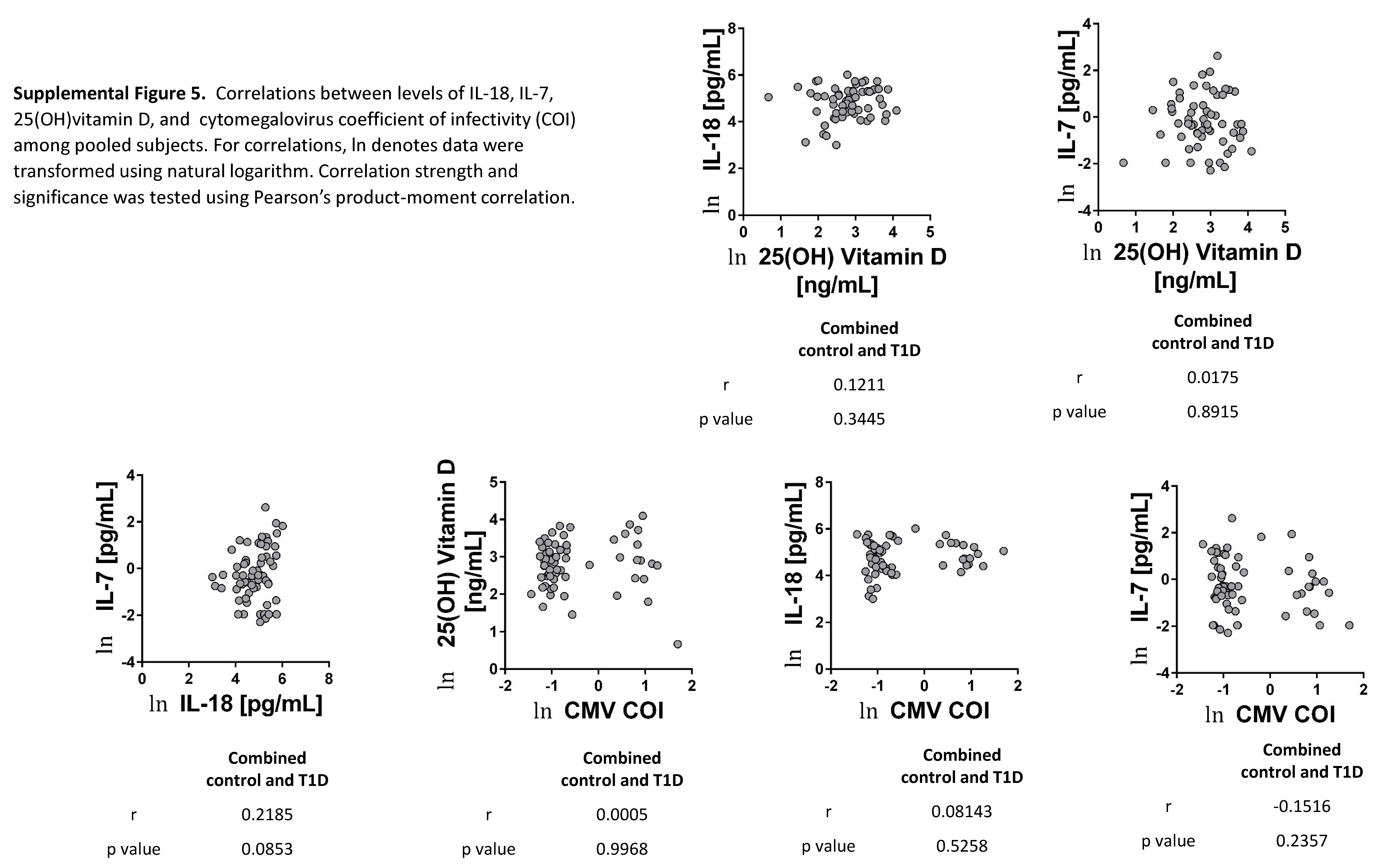

Supplement: Supplementary file 5 [file Image_5.TIF]

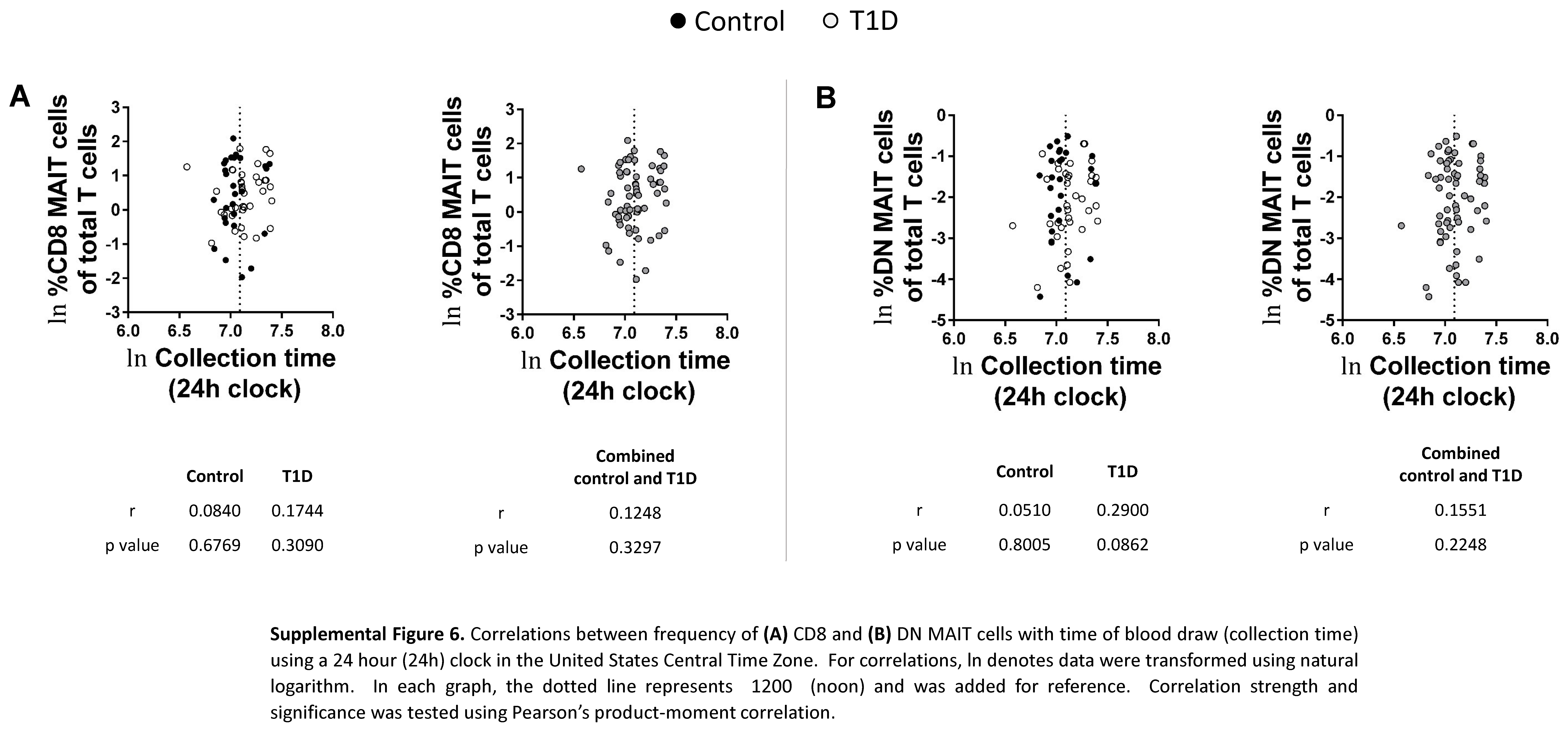

Supplement: Supplementary file 6 [file Image_6.TIF]
